# Factors associated with hemorrhagic fever with renal syndrome based maximum entropy model in Zhejiang Province, China

**DOI:** 10.3389/fmed.2022.967554

**Published:** 2022-10-05

**Authors:** Rong Zhang, Ning Zhang, Ying Liu, Tianxiao Liu, Jimin Sun, Feng Ling, Zhen Wang

**Affiliations:** ^1^Key Laboratory of Vaccine, Prevention and Control of Infectious Disease of Zhejiang Province, Department of Communicable Disease Control and Prevention, Zhejiang Provincial Center for Disease Control and Prevention, Hangzhou, China; ^2^Puyan Street Community Health Service Center of Binjiang District, Hangzhou, Zhejiang, China; ^3^School of Science and Technology, University of Tsukuba, Tsukuba, Japan

**Keywords:** MaxEnt, HFRS, socio-economic factors, ecological data, meteorological data

## Abstract

**Background:**

Hemorrhagic fever with renal syndrome (HFRS) is a serious public health problem in China. The geographic distribution has went throughout China, among which Zhejiang Province is an important epidemic area. Since 1963, more than 110,000 cases have been reported.

**Methods:**

We collected the meteorological factors and socioeconomic indicators of Zhejiang Province, and constructed the HFRS ecological niche model of Zhejiang Province based on the algorithm of maximum entropy.

**Results:**

Model AUC from 2009 to 2018, is 0.806–0.901. The high incidence of epidemics in Zhejiang Province is mainly concentrated in the eastern, western and central regions of Zhejiang Province. The contribution of digital elevation model ranged from 2009 to 2018 from 4.22 to 26.0%. The contribution of average temperature ranges from 6.26 to 19.65%, Gross Domestic Product contribution from 7.53 to 21.25%, and average land surface temperature contribution with the highest being 16.73% in 2011. In addition, the average contribution of DMSP/OLS, 20-8 precipitation and 8-20 precipitation were all in the range of 9%. All-day precipitation increases with the increase of rainfall, and the effect curve peaks at 1,250 mm, then decreases rapidly, and a small peak appears again at 1,500 mm. Average temperature response curve shows an inverted v-shape, where the incidence peaks at 17.8^°^C. The response curve of HFRS for GDP and DMSP/OLS shows a positive correlation.

**Conclusion:**

The incidence of HFRS in Zhejiang Province peaked in areas where the average temperature was 17.8^°^C, which reminds that in the areas where temperature is suitable, personal protection should be taken when going out as to avoid contact with rodents. The impact of GDP and DMSP/OLS on HFRS is positively correlated. Most cities have good medical conditions, but we should consider whether there are under-diagnosed cases in economically underdeveloped areas.

## Introduction

Hemorrhagic fever with renal syndrome (HFRS) is a rodent viral disease caused by hantaviruses (HVs), which are distributed in Eurasia and have a case fatality rate of 5–15%; HVs can also cause hantavirus cardiopulmonary syndrome (HCPS), which develop mainly in the Americas and have a case fatality rate of up to 40% for HCPS (1). Since there are no specific drugs, HFRS and HCPS can only be treated with symptomatic support. HFRS caused by Hantaan (HTNV) and Seoul (SEOV), HTNV and SEOV which belongs to genus in Orthohantavirus family Hantaviridae, order Bunyavirales (2). Whole virus-inactivated vaccines against HTNV or SEOV are currently licensed in Korea and China, but the protective efficacy of these vaccines is uncertain (1).

HFRS is a serious public health problem in China. The epidemic reached the peak in 1986, with a total of 115,804 cases reported. As government launched a series of disease control and prevention measures, the incidence began to decrease (3). HFRS was first observed in the Heilongjiang Province of China in the 1930s. It remained poorly understood until 1978 when Hantaan Virus and its reservoir *Apodemus agrarius* were discovered by Lee et al. (4). During 2006–2012, 77,558 human cases and 866 fatal cases of HFRS were reported, with an average annual incidence rate of 0.83 per 100,000 and a case fatality rate of 1.13%. About 84.16% of the total cases were concentrated in 9 provinces, with the highest incidence in spring and autumn/winter (5).

Zhejiang Province is an HFRS endemic region, and the first case was reported in 1963. From 1963 to 2020, the morbidity and mortality rates decreased significantly, however, the geographical distribution of endemic areas has been expanding to all of Zhejiang Province (6).

Studies had shown hantavirus infection dynamics: changes in climate (7–9), environmental condition affect the risk of zoonotic transmission via changes in reservoir dynamics (8); nephropathia epidemica is more likely to occur with intense vegetation activity, soil with low water content condition (10). Previous analyses in China suggest rainfall, mouse density and autumn crop yield are correlated with the incidence of HFRS (11). A study in Xi’an, China showed HFRS is correlated to rainfall, rodent density and lags of temperature (12). Another research observed HTNV stabilities and results show at 4 degrees wet conditions particularly, HTNV is detectable after 96 days, and sensitive to drying (13).

## Materials and methods

### Data collection and case definition

The data from 2009 to 2018 on HFRS cases were obtained from the Chinese Notifiable Disease Reporting System. Information of HFRS cases includes age, gender, residential address and date of illness onset. According to the health industry standard of the People’s Republic of China for diagnostic criteria of HFRS, HFRS cases were classified as suspected cases, clinically diagnosed cases and confirmed cases (6). Zhejiang Province is located on the southeast coast of China, with 11 cities and 90 county (Supplementary Figure 1).

Meteorological data (DEM01-11) were obtained from China Meteorological Data Sharing Service System.^1^ It included sunshine hours, average relative humidity, average land surface temperature, 20–8 precipitation, 8–20 precipitation, 20–20 precipitation, average air pressure, average air temperature, daily maximum temperature, average wind speed and maximum wind speed. Layers for yearly average meteorological data from 2009 to 2018 were generated using the kriging interpolation method with ArcGIS 10.2.

The socio-economic factors and ecological data used in this study were obtained from the Resource and Environmental Science Data Center of the Chinese Academy of Sciences.^2^ They included normalized difference vegetation index (NDVI), enhanced vegetation index (EVI), Annual NDVI (aNDVI); clay, sand, silt; gross domestic product (Zjgdp), and digital elevation model. Digital elevation model (DEM) can be regarded as an image, a typical raster data obtained by sampling the image plane coordinates and height. Defense Meteorological cSatellite Program Nighttime Lighting Index (DMSP/OLS), DMSP/OLS imageries were acquired by the Defense Meteorological Satellite, mainly used for urban expansion research. We used ArcGIS 10.2 to set the grid for layers of different variables to the same geographic boundaries and cell sizes to extract data for Zhejiang Province.

### Statistical methods

The maximum entropy principle is to predict the unknown information of a target area by incomplete known information. Using known species distribution and ecological environment data, the non-random relationship between environmental characteristics and species distribution in the known species distribution area is studied to find the probability distribution with maximum entropy as the optimal distribution to predict the suitable habitat for a species (14).

The maximum entropy model (MaxEnt) is a valuable software for ecological niche models (ENM) study to estimate the habitat suitability of a species through occurrence data and a set of environmental variables (15). It is well documented that MaxEnt has great advantages in epidemiological studies of natural epidemics, detecting the main meteorological factors influencing the high incidence of infectious diseases (16–18) and predicting the impact of future climate change on local species (19, 20). MaxEnt is a machine learning algorithms based on the maximum information entropy to construct the model. The prediction structure is accurate, and its response curve depends on the data characteristics. It can obtain more accurate results with only a small number of sample data (21).

To select the best model, we consulted with epidemiologists on which factors might be associated with the occurrence of HFRS and performed cross-correlation analysis to effectively identify multicollinearity. We selected a validity variable from the variables of multicollinearity. Different regularization multipliers (RM) were adjusted in MaxEnt, then we selected the features that contributed most to the model, thus reducing model overfitting (20, 22).

In this study, ecological niche models of HFRS were constructed using Maxent 3.4.1. Spatial distribution of HFRS cases from 2009 to 2018 was dependent variable; meteorological factors, socio-economic factors and ecological data were independent variables. In our modeling, 75% of the data are randomly selected as the training set and the remaining 25% as the test set. The stability of the model is verified by the (cross-validation) method, and the result of the average of 10 modeling repetitions is used as the model result (21). In the parameter setting, Regularization multiplier was set as 1, Replicate type was set as bootstrap, Replicates was set as 10, Max number of background points was set as 10,000, Max iterations was set as 500. In general, AUC values < 0.7 are considered low accuracy, 0.7–0.9 are considered useful for applications, and >0.9 are considered high accuracy (23, 24).

## Results

From 2009 to 2018, the cumulative number of HFRS reported cases in Zhejiang Province was 4240, The annual numbers of HFRS cases in each year during 2009–2018 were 438, 458, 541, 501, 526, 385, 362, 349, 353, and 327. Cases were reported in 80% of counties in Zhejiang province, and the number of counties reporting cases each year from 2009 to 2018 were 62, 56, 59, 61, 65, 63, 61, 62, 71, and 63.

**FIGURE 1:**
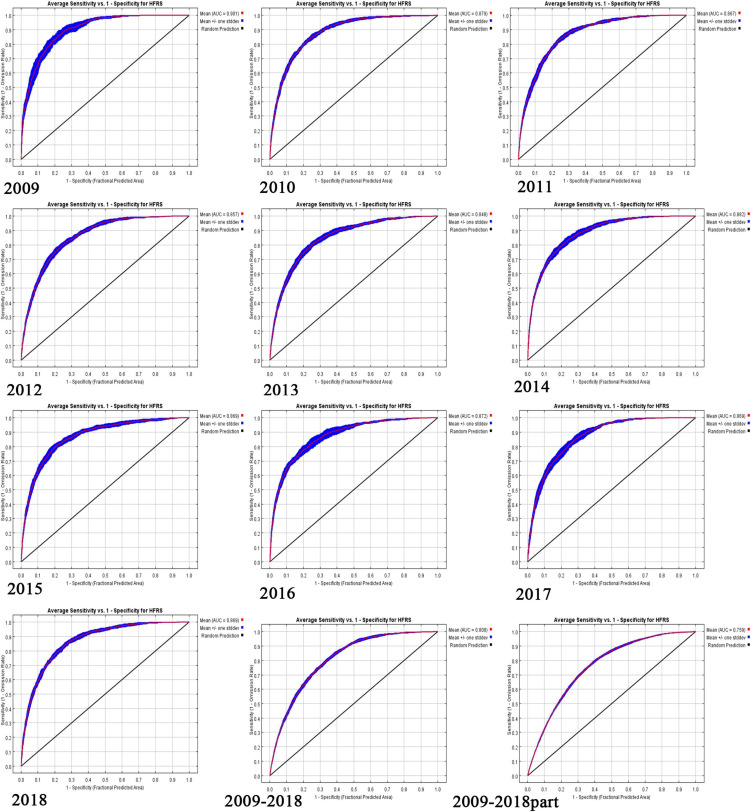
The receiver operating characteristic (ROC) curve for HFRS from 2009 to 2018.

Figure 1 is the receiver operating characteristic (ROC) curve for the again averaged over 10 replicate, and AUC for different years were given in ENMs. The AUCs for 12 models, 2009, 2010, 2011, 2012, 2013, 2014, 2015, 2016, 2017, 2018, 2009–2010 average of 2009–2018 were 0.901, 0.879, 0.867, 0.857, 0.848, 0.882, 0.869, 0.872, 0.869, 0.869, 0.808, and 0.759. (Part of 2009–2018*: We selected all variables that contributed more than 5% in the above 11 models to be included in this model).

**FIGURE 2:**
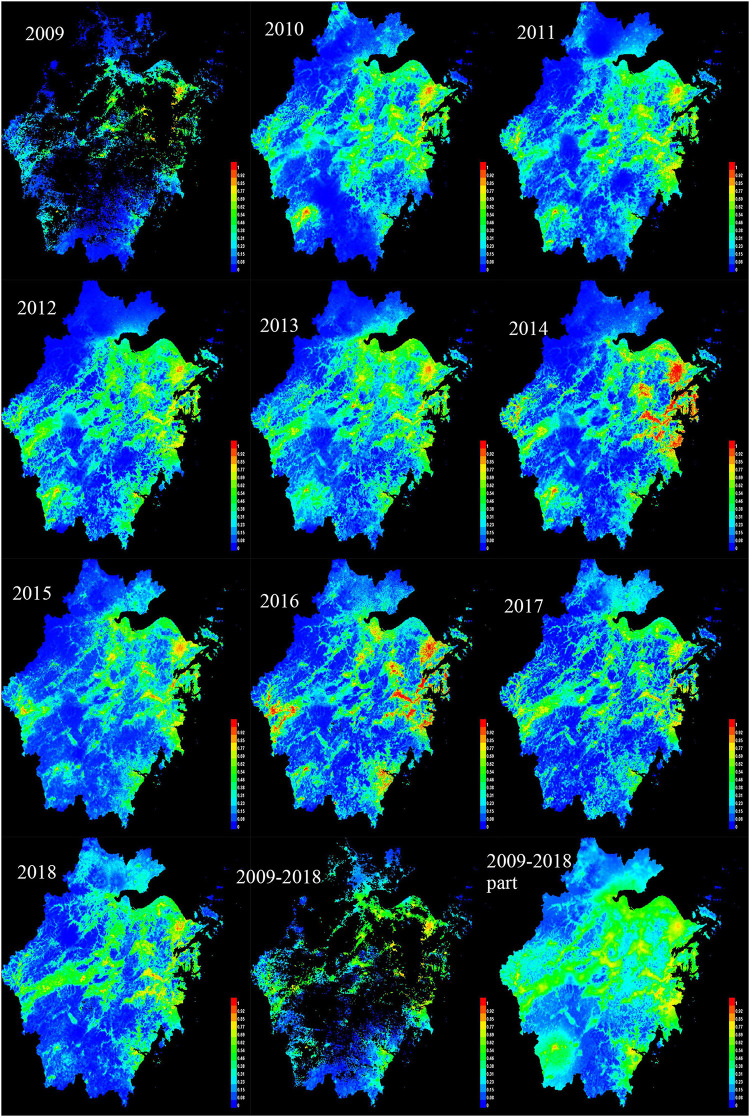
The predicted potential risk map of HFRS from 2009 to 2018 in Zhejiang Province.

Figure 2 shows the predicted potential risk map of HFRS from 2009 to 2018 in Zhejiang Province.

The legend on the right side of the picture shows the different risk levels in red, orange, green and blue in descending order, with red representing the highest risk and, and blue representing the lowest risk. It can be concluded from the above risk map that the high incidence of epidemics in Zhejiang Province is mainly concentrated in the eastern and western regions, as well as in the central region.

Table 1 shows the contribution of each variable to the final training MaxEnt model in different years used in this study, showing the mean and average of the 10 replicate runs. According to Table 1, it can be seen that zjdem, average temperature and GDP contribute the most to the MaxEnt model constructed in Zhejiang Province.

**TABLE 1:** The contribution of each variable to the final training MaxEnt model.

Variables	2009 (%)	2010 (%)	2011 (%)	2012 (%)	2013 (%)	2014 (%)	2015 (%)	2016 (%)	2017 (%)	2018 (%)	2009–2018 (%)
zjdem	15.75	6.38	7.56	4.22	18.43	13.06	11.00	10.19	12.16	26.00	20.98
demo8	19.65	16.25	6.53	8.20	7.11	10.26	12.30	9.67	6.26	9.20	14.63
gdp	9.79	7.53	12.94	20.09	16.79	13.32	11.09	19.11	21.25	8.55	11.81
demo3	8.57	11.01	16.73	3.42	14.89	10.98	11.19	3.29	9.69	1.67	10.79
zjdmsp	6.02	17.80	13.96	13.05	8.61	11.51	14.31	11.26	2.92	13.61	9.05
demo4	0.90	1.16	3.11	19.22	11.08	0.88	2.93	1.35	4.51	0.76	9.00
demo5	6.22	11.12	0.69	6.90	0.79	13.83	2.80	2.30	5.90	3.04	5.95
demo7	1.75	1.73	1.42	2.33	3.80	1.49	3.03	4.53	2.04	2.58	3.20
ndvi	4.76	2.46	3.82	3.59	2.39	2.25	4.40	4.05	13.05	3.44	2.76
demo2	3.40	6.21	8.05	5.62	2.67	10.82	4.79	10.02	3.33	3.57	2.75
andvi	2.51	5.04	4.57	4.41	2.55	3.07	3.33	6.08	7.21	4.52	2.12
demo9	1.16	2.41	1.38	0.71	1.72	1.82	1.33	0.99	1.60	1.46	1.75
evi	1.82	1.33	2.32	1.57	2.79	2.32	6.55	2.01	2.42	1.97	1.59
demo1	3.09	3.64	2.08	1.19	2.49	0.89	0.69	1.81	1.49	13.58	1.43
demo6	9.23	1.06	12.09	2.07	0.92	0.98	4.39	4.84	1.38	0.99	0.37
demo10	1.34	1.82	0.64	0.64	0.47	0.94	1.99	0.96	0.58	0.54	0.56
demo11	0.94	1.66	0.54	0.57	0.47	0.39	1.85	5.57	1.71	2.48	0.55
sand	1.71	0.35	0.42	0.36	0.49	0.52	0.53	1.14	1.00	0.45	0.09
silt	0.60	0.80	0.32	1.22	1.12	0.48	1.16	0.41	0.84	0.70	0.40
clay	0.82	0.24	0.82	0.61	0.40	0.18	0.37	0.47	0.67	0.88	0.23

DEM that made the greatest contribution varied significantly by years, ranging from 4.22 to 26.0% from 2009 to 2018. Average temperature had the second highest contribution, ranging from 6.26 to 19.65% during 2009–2018. The third highest contribution is gross domestic product, contribution during 2009–2018 ranging from 7.53 to 21.25%. The contribution of average land surface temperature ranked fourth, and it fluctuates greatly from year to year, with the highest being 16.73% in 2011, but the lowest being only 1.67% in 2018. In addition, the average contribution of DMSP/OLS, 20-8 precipitation and 8-20 precipitation were all in the range of 9%, even though the contribution of DMSP/OLS was as high as 17.8% in 2010.

**FIGURE 3:**
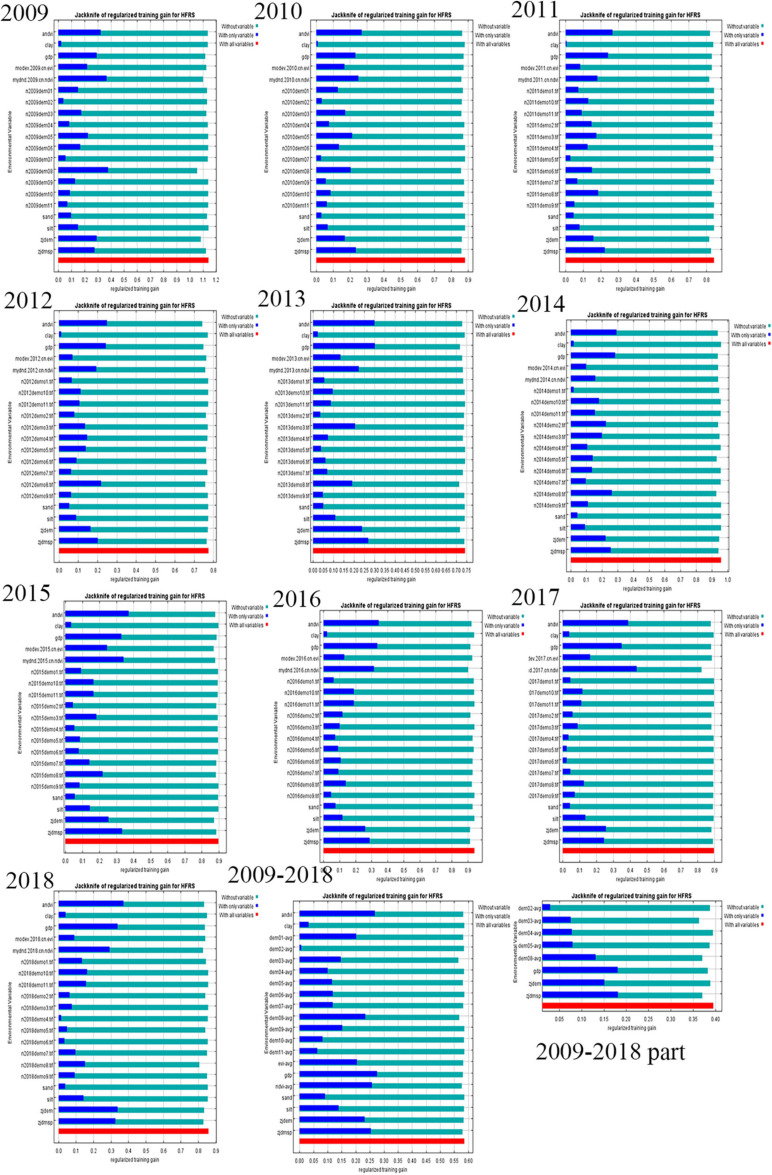
The results of the jackknife test of variable importance.

The following picture shows the results of the jackknife test of variable importance. In Figure 3, the dark blue bar is the separate contribution of the variable and the light blue bar is the contribution that does not include that variable.

**FIGURE 4:**
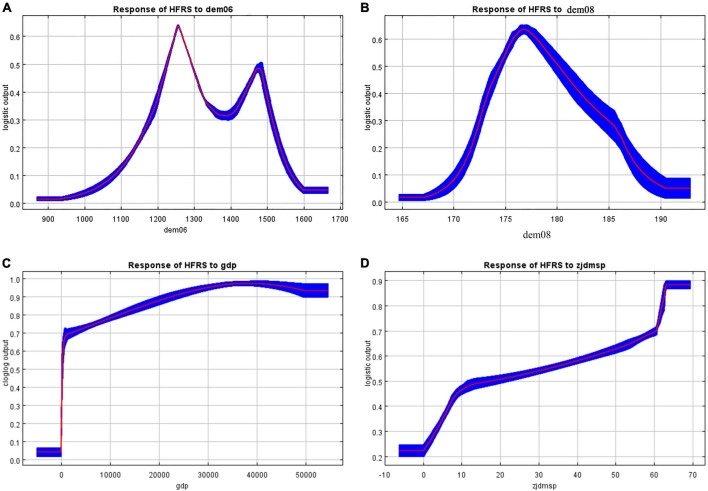
The response curves contributed to the MaxEnt model of HFRS.

Figure 4 shows the response curves for the 4 variables that contributed the most to this study after excluding the variables that contributed less than 5%.

Figure 4A shows the effect curve of All-day Precipitation, and it can be seen that the incidence of HFRS increases with the increase of rainfall, and the effect curve peaks at 1,250 mm, then decreases rapidly, and a small peak appears again at 1,500 mm; through Figure 4B, we can see that average temperature response curve shows an inverted v-shape, the incidence of HFRS increases with the increase of average temperature, and the incidence peaks at 17.8^°^C, followed by a rapid decrease; through Figure 4C, we can see the response curve of HFRS and GDP showing a positive correlation—the incidence increases with the increase of GDP; through Figure 4D, we can see the response curve of DMSP/OLS Nighttime Light Data (DMSP/OLS), also show positive correlation—the incidence rate increases with the increase of DMSP/OLS.

## Discussion

The advantage of the maximum entropy model is that it is based on the prediction of positive points, avoiding the problem of missing negative data, and it predicts the probability magnitude of risk, which is more widely used in the field of infectious diseases. The prevention and control of any infectious disease cannot be separated from three major elements: infectious source, transmission route and susceptible population. HFRS is a natural epidemic disease, and the influence of meteorological factors on natural epidemic disease needs to be considered from various aspects.

Yuan et al. constructed a maximum entropy model with 16 environmental factors, and then they considered NDVI, rainfall variance and elevation as the main environmental factors affecting landslide hazard (25). Sun et al. constructed ENM of severe fever with thrombocytopenia syndrome (SFTS) using MaxEnt, and they found yearly average temperature, altitude, yearly average relative humidity and yearly accumulated precipitation accounted for 94.1% contribution for ENM (17). Fang et al. found that HFRS incidence in Shandong Province is mainly associated with the seasonal environmental variables: temperature, precipitation and humidity (26). Our study confirmed the contribution of mean temperature to the incidence of HFRS in Zhejiang Province ranged from 6.26 to 19.65% in 2009–2018.

Firstly, temperature have impact on people’s willingness of travel, and the probability of HFRS infection will increase when people go out in favorable temperature and sunny weather (27); secondly, temperature will affect the reproduction and activities of host animals and vector organisms, and only when the density of host animals and vector organisms reaches a certain level, human can be infected when they go out (27).

We likewise found some fluctuations in the effect of all-day precipitation on the onset of HFRS, with the effect curve peaking at 1,250 mm and then declining rapidly, with another small peak at 1,500 mm. This is consistent with many studies in which there is a certain lag in the onset of HFRS by rainfall. Studies have confirmed that there is a lag effect of meteorological factors on the onset of HFRS, and the conclusions obtained from different study areas vary from several weeks to several months (27–29). Cao et al. found that the effect of temperature on HFRS varies widely among regions with different temperature zones, ranging from a lag of 1 month in temperate regions to 3 months in subtropical regions (29).

Our findings concern seasonal patterns of HFRS in Zhejiang Province of China that there are two peak incidences of HFRS, one from May to June and the other from November to January (6). The host of SEOV- *Rattus norvegicus* is more common in urban while the host of HTNV—*Apodemus agrarius* is more likely to inhabit rural areas (30). Zhejiang Province has shifted from a single peak incidence in winter in the early stage to two peak incidences in winter and spring at this stage, based on the reasons described below, which is also a renewal of previous concepts. The first reason is that the main host animal of the spring peak is the *Rattus norvegicus* (26), and the main host animal in the urban residential area is the *Rattus norvegicus*, which lives very closely with humans, resulting in an increase in the incidence of urban areas. Secondly, the medical conditions of urban residents are significantly better than those in rural areas, and their medical security and transportation conditions have played a key role (31).

Our results found that the incidence of HFRS shows a positive correlation with GDP and DMSP/OLS response curves, which is consistent with our earlier findings and could further explain why Ningbo city has the province’s most number of cases, compared with other cities. GDP (32) and DMSP/OLS are socioeconomic indicators, and many scholars have used DMSP/OLS to study urban development and agreed that DMSP/OLS has strong potential for urbanization research (33, 34).

DMSP/OLS images can be used as a characterization of human activities and become a good data source for human activity monitoring studies. One of the main advantages of this data is that it does not depend on high spatial resolution, which is usually around 1 km, so it is easier to process DMSP/OLS data; another important feature is that it covers information closely related to the distribution of factors such as population and city layout, like traffic roads and residential places (33).

Zhejiang Province is mainly concentrated in the eastern and western regions, which is consistent with the results of our earlier study (6). The city with the highest incidence rate in Zhejiang Province is Ningbo, which is inseparable from the ability of local medical institutions to detect and diagnose diseases (35). Ningbo is a sub-provincial city with developed economy, convenient transportation and high level of medical services. Due to the rapid development of Ningbo, a large number of rural areas have been merged into the city, and the living environment of the urban-rural integration area is not perfect, which is also one of the reasons for the high incidence of local diseases.

The peak incidence in summer mainly is related to indoor infection caused by the breeding of domestic rodent, and the peak incidence in winter is related to exposure to wild rodent in the field (36). The HFRS monitoring in Zhejiang Province found that *Apodemus agrarius* accounted for 59.71% of the total number of wild rodent captured, and *Rattus norvegicus* accounted for 10.77% of the total number of domestic rodent captured (37). The clinical symptoms and outcomes of HTNV-caused HFRS are usually more serious than those of SEOV-caused (38).

Our study found that the incidence of HFRS varies greatly among regions in Zhejiang Province, but Zhoushan city always has the lowest incidence in the province. Another study found that the incidence of SFTS in Zhoushan city ranks among the top in the province, and we speculate that local rainfall and humidity have a certain relationship with this incidence (39). The MaxEnt model has great advantages in predicting the survival of endangered species. Scholars have used the optimized MaxEnt model to predict the distribution of Quasipaa boulengeri in different provinces in China is important environment variables (40).

The advantage of this study is that two environmental variables and two economic indicators associated with the incidence of HFRS in Zhejiang Province were found by incorporating meteorological factors and socioeconomic indicators. The ecological locus model with maximum entropy explored the incidence risk areas and provided a reference basis for the rational allocation of medical resources in the province.

This study has the following disadvantage: the international algorithms of ecological niche models are complex and diverse, and we only used the most widely used and stable maximum entropy algorithm to analyze the HFRS epidemic in Zhejiang Province, thus we are unable to achieve an in-depth and comprehensive analysis. Since only some counties in Zhejiang Province are under surveillance, the lack of molecular epidemiological analysis of the impact is also a major shortcoming of the study.

## Conclusion

Although many models have been used for the study of risk factors of infectious diseases, the present study confirmed that the maximum entropy model for the study of risk factors of HFRS in Zhejiang Province was in good agreement with the results of previous studies. In summary, the incidence of HFRS peaks in areas where the average temperature is 17.8 degrees Celsius, which reminded us that in areas where temperature is suitable, personal protection should be taken when going out to avoid contact with rodents. The incidence of HFRS by rainfall is more complicated and fluctuates to a certain extent, which is also consistent with the conclusions of most studies, and there is a certain lag in the impact on the disease. The impact of GDP and DMSP/OLS on HFRS is positively correlated. Most cities have good medical conditions, but it also reminds us whether there are under-diagnosed cases in economically underdeveloped areas.

## Data availability statement

The original contributions presented in this study are included in the article/Supplementary material, further inquiries can be directed to the corresponding author/s.

## Ethics statement

This study was reviewed and approved by the Ethics Committee of the Zhejiang Provincial Center for Disease Control and Prevention (No. 2020-021). All data used in this study followed the Law of the Prevention and Treatment of Infectious Diseases in the People’s Republic of China. As only surveillance information was analyzed, this study did not involve human research, informed consent was waived by the Ethical Institutional Review Board.

## Author contributions

RZ: conceptual, methodology, writing—original draft preparation and funding acquisition. NZ: analysis and modeling. YL: software. TL: improve the language. JS: project administration and writing and editing. FL: writing—review and supervision. ZW: supervision. All authors contributed to the article and approved the submitted version.

## Footnotes

1
http://data.cma.cn/

2
http://www.resdc.cn
