# Supplementary figures and images for: Factors associated with hemorrhagic fever with renal syndrome based maximum entropy model in Zhejiang Province, China
Source: Front Med (Lausanne). 2022 Oct 5;9:967554. doi: 10.3389/fmed.2022.967554 (PMC9579348; doi:10.3389/fmed.2022.967554)

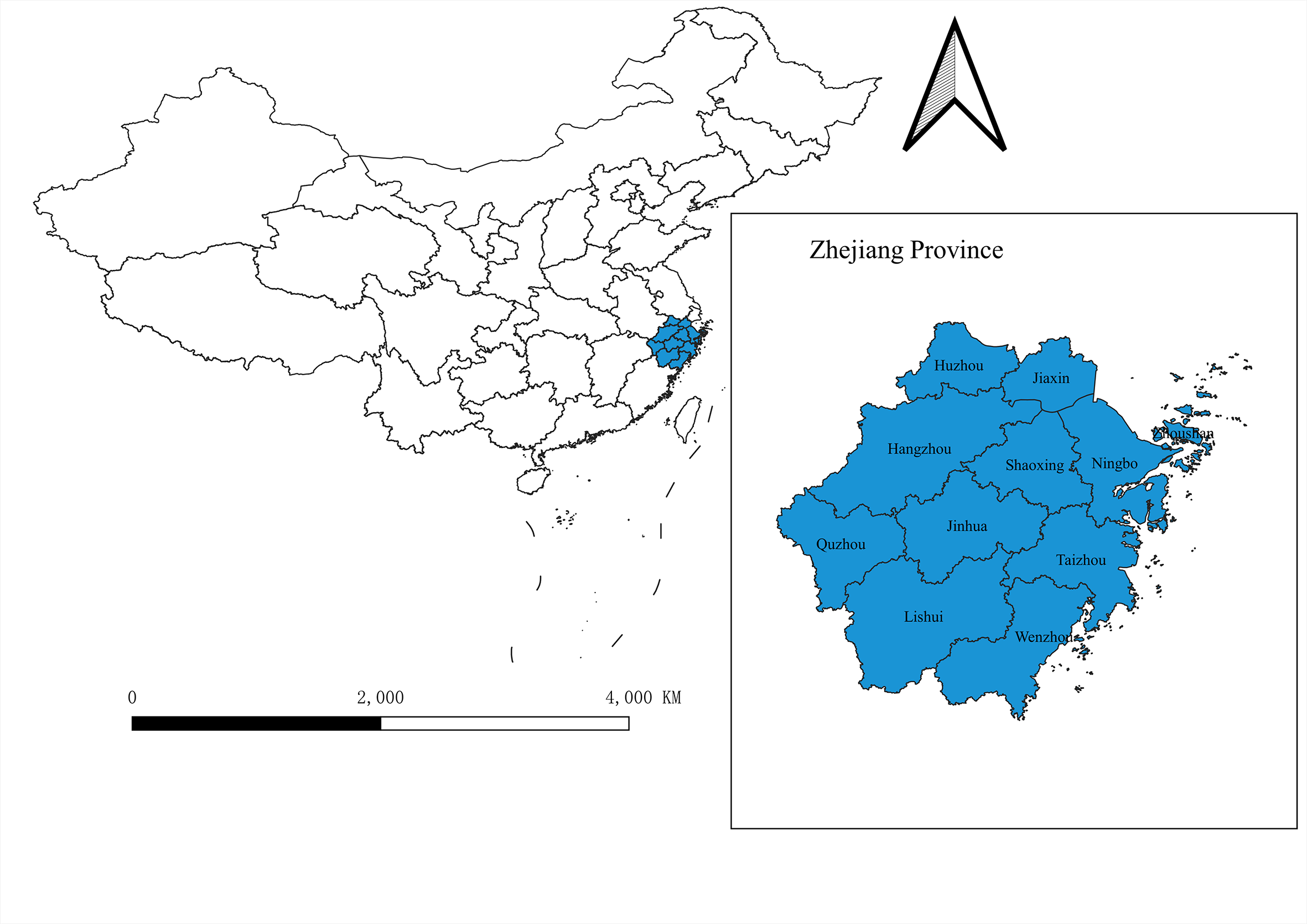

Supplement: Supplementary file 1 [file Image_1.TIFF]
